# D-Ribose Glycation of Human High-Density Lipoprotein: Structural and Functional Alterations

**DOI:** 10.3390/ijms27104370

**Published:** 2026-05-14

**Authors:** Camilla Morresi, Valeria Di Tomaso, Giovanni Ricci, Gianna Ferretti, Tiziana Bacchetti

**Affiliations:** 1 Department of Life and Environmental Sciences, Polytechnic University of the Marche, 60131 Ancona, Italy; c.morresi@staff.univpm.it (C.M.); s1118680@studenti.univpm.it (G.R.); 2 Translational Hematology Unit—Biosciences Laboratory, IRCCS Istituto Romagnolo per lo Studio dei Tumori “Dino Amadori” (IRST), 47014 Meldola, Italy; valeria.ditomaso@irst.emr.it; 3 Department of Clinical Sciences and Odontostomatology, Faculty of Medicine, Polytechnic University of the Marche, 60131 Ancona, Italy

**Keywords:** non-enzymatic glycation, high-density lipoproteins, D-ribose, oxidative stress

## Abstract

Glycation of biomolecules leads to the formation of advanced glycation end products (AGEs) and is implicated in molecular mechanisms of human chronic diseases. We compared the glycation properties of D-ribose and methylglyoxal on human high-density lipoprotein (HDL). The increase in fluorescent AGEs in HDL samples treated with methylglyoxal confirms that HDLs are sensitive to glycation treatment. Our results demonstrated that even D-ribose glycates HDL as shown by changes induced by D-ribose on HDL apoprotein. Biochemical markers of lipid and protein oxidative damage were also evaluated. The increase in protein carbonyl contents and thiobarbituric acid reactive substances (TBARS) demonstrates a glyco-oxidative stress occurs in D-ribose treated HDL. In addition, HDL treated with D-ribose showed a significant decrease in the activity of the enzyme paraoxonase 1 and an increased HDL redox activity. These data demonstrate that D-ribose-induced glycation of HDL may impair lipoprotein functionality and may contribute to molecular mechanisms of dysmetabolic diseases.

## 1. Introduction

High-density lipoproteins (HDLs) exert key physiological roles, modulate inflammation and regulate cholesterol uptake and cellular metabolism. The pleiotropic roles are due to the complex lipid and protein composition of HDL [1,2,3]. Among proteins associated with the HDL surface, there is the enzyme paraoxonase 1 (PON1). PON1 is included in a family of calcium-dependent enzymes which exert anti-inflammatory and antioxidant and properties [4,5,6]. Several in vitro and ex vivo experiments have demonstrated evidence that compositional alterations convert HDL to dysfunctional lipoproteins. Dysfunctional HDLs lose their protective properties and appear involved in molecular mechanisms of dysmetabolic diseases such as diabetes and coronary artery disease (CAD) [4,7,8,9,10,11]. Among compositional changes which contribute to dysfunctional HDL there are glycation and glyco-oxidative stress. Glycation of several plasma proteins including apoprotein AI (Apo-AI) and HDL occurs in diabetes in vivo and contributes to a decrease in HDL protective roles [11,12,13,14,15,16]. Several metabolites including glucose, fructose and other carbonyls such as methylglyoxal (MGO) react with plasma proteins, lipids or apoproteins of lipoproteins and contribute to their glycation [17,18]. Previous studies have shown that glycation reaction occurs between free carbonyl groups of reducing sugars and free amino groups of proteins and other biomolecules. A series of rearrangements, cyclization and dehydration of early glycation products leads to the formation of advanced glycation end-products (AGEs) [19]. The reactions involving glycation and oxidation of HDL are believed to contribute to the development of atherogenesis and AGEs are described in the molecular mechanisms of diabetes and age-related diseases [20,21]. HDL glycated in vitro in different experimental conditions represent useful models to investigate the biochemical and functional changes in HDL in diabetes and other human diseases. For instance, glycated HDL have a lower ability to uptake cholesterol from membranes [22] and exert a lower antioxidant effect [14,15,16,17]. Levels of glycated HDL are higher in diabetic patients compared to normal subjects, even in the presence of good glycemic control [14,23]. Therefore, great attention is devoted to molecules able to glycate lipoproteins and to the study of structural and metabolic alterations.

Recent studies have shown that D-ribose, the more stable form of the two enantiomers (L-ribose and D-ribose), causes glycation of plasma proteins and protein aggregation producing AGEs that lead to cell dysfunction and death and alterations of their functional activities [24,25,26,27,28,29,30,31,32,33,34,35,36,37,38]. A potential link between alterations of D-ribose metabolism and some clinical conditions, such as type 2 diabetes mellitus and cognitive dysfunction, has been recently shown [39]. A significant increase in urinary D-ribose levels in individuals with diabetes compared to healthy individuals has been demonstrated [40,41,42] and it has been proposed that alterations of D-ribose metabolism could be involved in the molecular mechanisms of human dysmetabolic diseases. It has been shown that low-density lipoproteins (LDL) are susceptible to glycation triggered by D-ribose [25,26,29]. No report has been previously published on the effect of D-ribose-induced glycation (ribosylation) on HDL composition and functions.

The main aim of the study was to investigate the susceptibility of HDL to D-ribose glycation, and we characterized the modifications of HDL particles using various spectroscopic techniques. We also compared the alterations triggered by D-ribose on HDL with respect to MGO, a well-known potent glycating agent. Moreover, the effect of D-ribose glycation on HDL function has been studied. In particular, the activity of the enzyme PON1, associated with the HDL surface and HDL antioxidant properties, has been studied in D-ribose-treated HDL.

## 2. Results

### 2.1. Advanced Glycation End-Products (AGEs)

The glycating properties of D-ribose were compared with those of MGO, a well-known potent glycating agent. As shown in Table 1, a significant increase in total fluorescent AGEs was realized in HDL treated with 0.1 mM MGO for 7 days. Using 1 mM MGO, fluorescent AGEs formation was significantly increased even after 3 days of treatment. A time- and concentration-dependent increase in total fluorescent AGEs, pentosidine and vesperlysine was observed after incubation of HDL with D-ribose. The results demonstrate, for the first time, that human HDL proteins are susceptible to D-ribose-induced glycation. The significant increase in fluorescent AGEs levels was observed in HDL incubated at the lowest D-ribose concentration of 20 mM for 7 days. At higher concentrations of D-ribose (40 mM), the results showed a twice and threefold increase after 3 days and 7 days of incubation, respectively. Further increases in AGEs levels were observed in HDL incubated with D-ribose 80 mM both at 3 and 7 days (Table 1).

### 2.2. Changes in HDL Apoprotein

**Figure 1:**
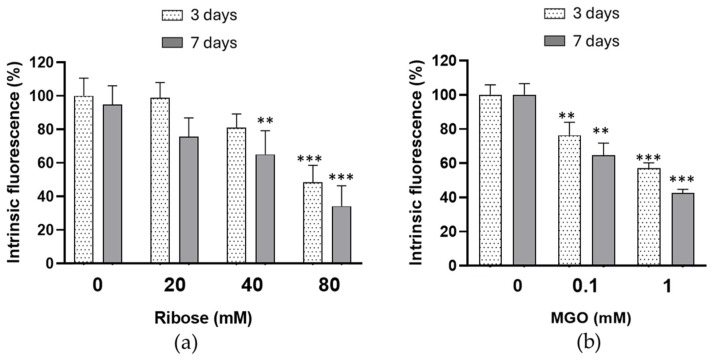
Intrinsic tryptophan fluorescence emission in HDL incubated in the absence (control HDL) or in the presence of increasing concentrations of D-ribose (**a**) or MGO (**b**) for 3 and 7 days. Data are reported as percentage of emission fluorescence intensity at λ = 340 compared to control HDL. Data are mean ± standard deviation (SD) of three independent experiments. ** *p* < 0.01, *** *p* < 0.001 vs. control HDL.

Changes in HDL apoprotein conformation related to D-ribose induced glycation have been investigated by the study of hyperchromicity and tryptophan (Trp) intrinsic fluorescence. As shown in Figure 1a, HDL treatment with D-ribose resulted in changes in the HDL absorption patterns with an increased absorption at 280 nm. Glycation of HDL proteins lead to an increase in their absorbance due to the formation of different glycation adducts, and such phenomenon is termed as hyperchromicity. The increase in hyperchromicity was significant in HDL incubated with D-ribose 40 mM and 80 mM for 3 and 7 days.

**Figure 2:**
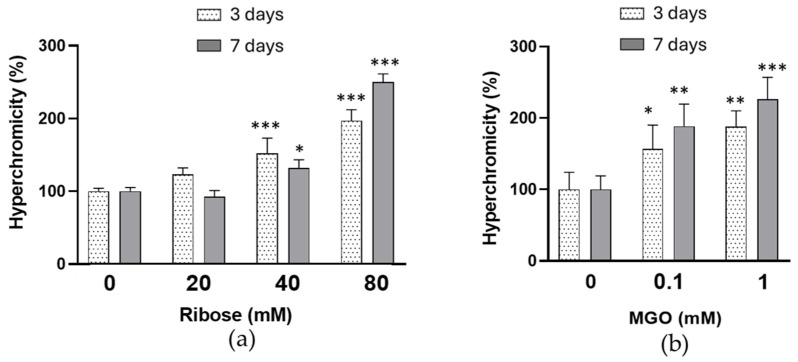
Hyperchromicity in HDL incubated in the absence (control HDL) or in the presence of increasing concentrations of D-ribose (**a**) or MGO (**b**) for 3 and 7 days. Data are reported as percentage of UV absorbance at 280 nm compared to control HDL. Data are mean ± standard deviation (SD) of three independent experiments. * *p* < 0.05, ** *p* < 0.01, *** *p* < 0.001 vs. control-HDL.

Modifications of intrinsic Trp emission fluorescence are a useful index of alterations in the conformation of HDL protein. As shown in Figure 2a, the emission fluorescence intensity at λ = 340 nm was decreased in HDL treated with D-ribose compared to control HDL. A 40% decrease in fluorescence intensity was observed in HDL glycated with 40 mM D-ribose for 7 days compared with control HDL. In HDL treated with 80 mM for 3 and 7 days, the emission fluorescence was 48.3 ± 5.8% and 34.1 ± 6.2%, respectively (Figure 2a).

A significant increase in hyperchromicity (Figure 1b) and a significant decrease in Trp fluorescence emission intensity (Figure 2b) were observed in HDL treated with MGO. For HDL treated with 1 mM for 3 and 7 days, the emission fluorescence was 57.0 ± 4.9% and 42.3 ± 6.5%, respectively, compared with control HDL.

### 2.3. Oxidative Damage

**Figure 3:**
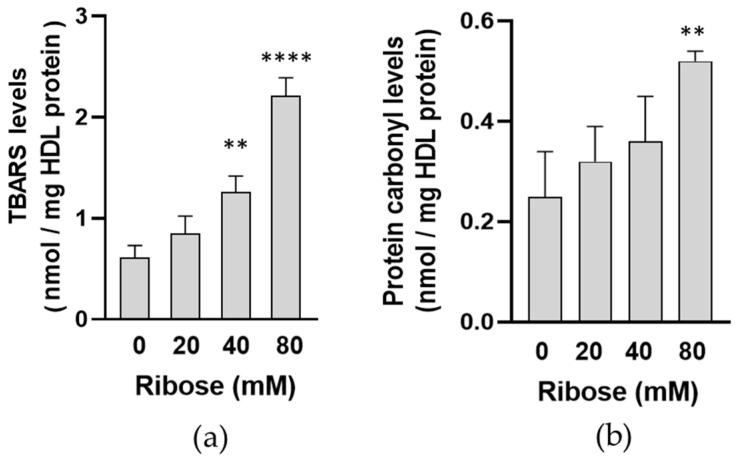
Levels markers of lipid peroxidation (**a**) and protein oxidation (**b**) in HDL incubated in the absence (control HDL) or in the presence of increasing concentrations of D-ribose for 7 days. Data are represented as mean ± standard deviation (SD) of three independent experiments. ** *p* < 0.01, **** *p* < 0.0001 vs. control HDL.

To further verify the effect of D-ribose treatment on HDL lipid and protein oxidative damage, the levels of thiobarbituric acid-reactive substances (TBARS) and of protein carbonyls were evaluated in control and D-ribose treated HDL. As shown in Figure 3a, the levels of TBARS in HDL treated with D-ribose 40 mM and 80 mM for 7 days were about two- and four-fold as compared to control HDL. A significant increase in the levels of protein carbonyls was observed in HDL treated with D-ribose at the higher concentration (Figure 3b).

### 2.4. HDL-Paraoxonase and HDL Redox Activity

**Figure 4:**
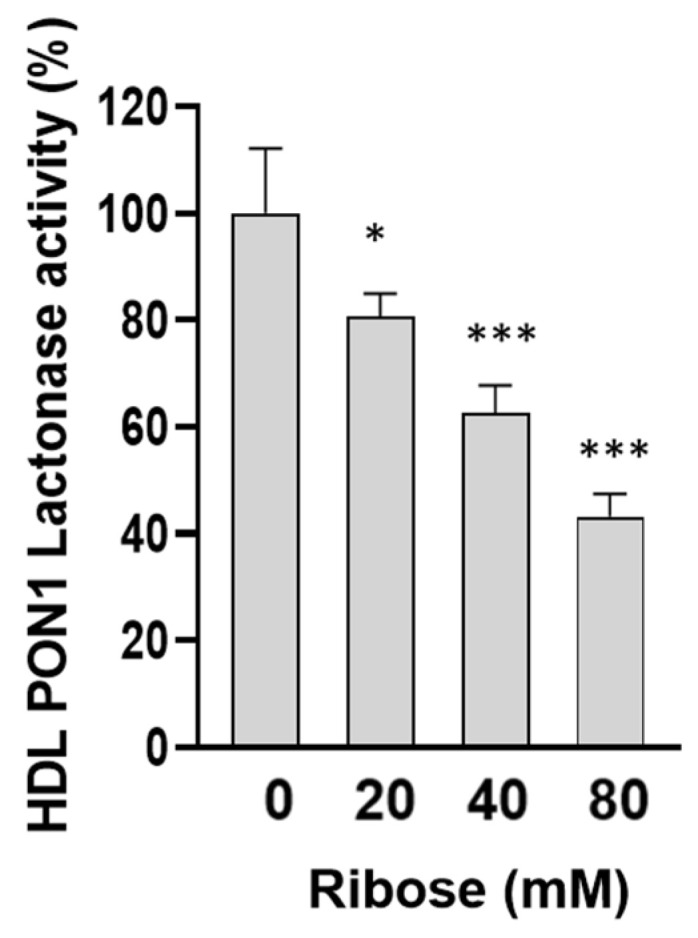
PON1 lactonase activity in HDL incubated in the absence (control HDL) or in presence of increasing concentrations of D-ribose for 7 days. Data are reported as percentage of lactonase activity compared to control HDL. Data are mean ± standard deviation (SD) of three independent experiments. * *p* < 0.05, *** *p* < 0.001 vs. control HDL.

To investigate whether D-ribose-induced glycation of HDL is associated with alterations of HDL functionality, the lactonase activity of the enzyme PON1 associated with HDL (HDL-PON) and the redox activity of HDL were evaluated. As shown in Figure 4, a significant decrease in HDL-PON activity was observed in HDL incubated with D-ribose for 7 days compared with control HDL.

**Figure 5:**
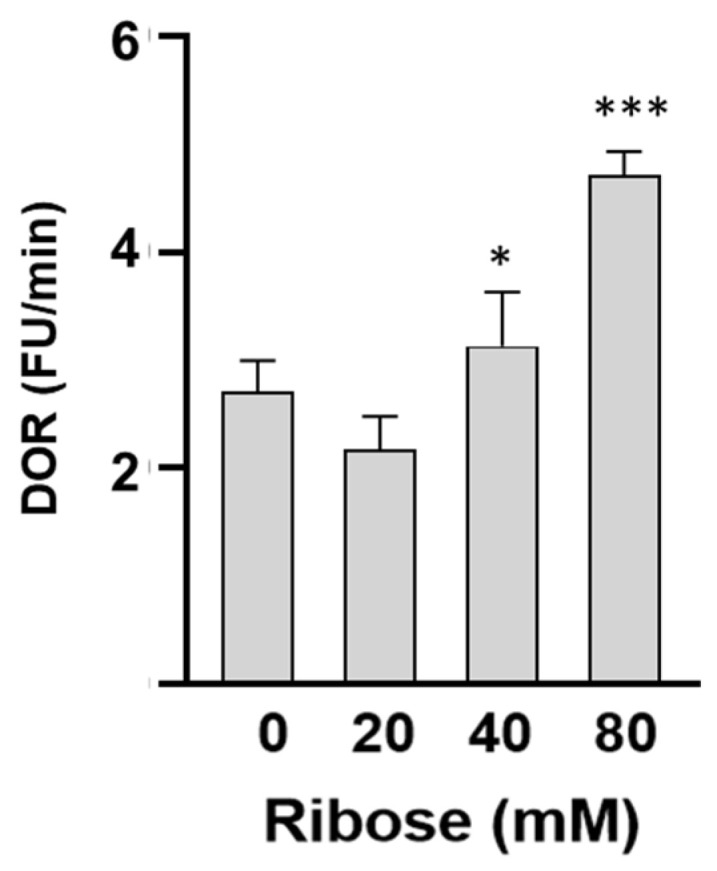
HDL redox activity in HDL incubated in the absence (control HDL) or in the presence of increasing concentrations of D-ribose for 7 days. Data are reported as percentage of DHR oxidation rate (DOR). Data are mean ± standard deviation (SD) of three independent experiments. * *p* < 0.05, *** *p* < 0.001 vs. control HDL.

HDL redox activity was assessed by measuring the increase in fluorescence of DHR oxidation over time (DOR). As reported in Figure 5, DOR values in HDL treated with D-ribose were significantly higher compared to control HDL. These results demonstrate that D-ribose-induced glycation increases the intrinsic ability of HDL to be oxidized.

These data are confirmed by the positive correlation established between DOR values and levels of TBARS evaluated in HDL treated in different experimental conditions (r = 0.66, *p* < 0.0001). A significant negative correlation was also established between PON1 lactonase activity and DOR values (r = −0.61, *p* < 0.0001) and TBARS values (r = −0.64, *p* < 0.0001) observed in HDL treated in different experimental conditions.

## 3. Discussion

D-ribose is produced by different pathways in living cells. For instance, D-ribose can be produced from glucose via the hexose monophosphate shunt and is released from nucleotides during metabolic turnover.

Previous in vitro studies have demonstrated that several proteins including hemoglobin, myoglobulin, albumin, insulin and LDL are glycated by D-ribose [24,25,26,27,28,29,30,31,32,33,34,35,36,37,38]. D-ribose glycated proteins show structural and functional alterations and are more susceptible to protein aggregation. In addition, the increase in AGEs during incubation of proteins with D-ribose is described more rapidly than glycation triggered by glucose [31,34,40]. Therefore, a growing attention is devoted to D-ribose glycation properties and effects on protein aggregation and oxidative stress. Among lipoproteins, previous studies have also demonstrated that HDL are susceptible to glycation in vivo [14,23] and in vitro by glucose or MGO [15,16,17]. Several alterations have been described in HDL glycated by glucose or MGO including a reduced removal of cholesterol from cells, and lower anti-inflammatory and antioxidant activity [15,16,17]. The effect of HDL glycation by D-ribose has not been performed till today.

The significant increase in total fluorescent AGEs shown in our experimental conditions after incubation of HDL with D-ribose confirms that human HDL proteins are susceptible to D-ribose-induced glycation. From the comparison with MGO emerges that D-ribose promotes total AGEs formation in HDL with different efficiencies. In fact, the glycating properties of MGO realize at lower concentrations and after shorter incubation times. D-ribose, although less reactive, still shows a remarkable glycating capacity leading to a progressive accumulation of fluorescent AGEs over time. Alterations of HDL proteins in D-ribose-incubated HDL are suggested by the significant increase in pentosidine and vesperlysine levels compared to control HDL. As shown in previous studies, pentosidine is considered a useful marker of protein glycation and is the result of cross-links between arginine and lysine residues [43]. Vesperlysine is described as a fluorescent derivative of sugar-derived cross-links of L-lysine [44]. Modifications of HDL apoprotein are confirmed by the decrease in tryptophan intrinsic fluorescence in D-ribose-treated HDL. Several studies have demonstrated that a decrease in the intrinsic fluorescence is related to a higher exposure of tryptophan residues to solvent molecules and represents an useful index of alteration in the tertiary structure of protein [45]. Therefore, our results suggest conformational changes in apoproteins in D-ribose-glycated HDL. The significant increase in hyperchromicity observed in HDL treated with D-ribose confirms conformational alterations of HDL apoproteins. In addition, the increase in protein carbonyl contents and TBARS, recognized as biomarker of oxidative stress, demonstrates a glyco-oxidative stress occurs in D-ribose-treated HDL. Our results are in agreement with previous studies which have confirmed that free radicals are formed during early and advanced glycation end-production [34,46,47]. Even glycation by D-ribose of LDL results in an increase in biomarker of oxidative stress [29].

As far as concerns the physio-pathological relevance of our results, other authors have shown that D-ribose-induced structural perturbations of plasma proteins are associated with functional alterations [24,25,26,27,28,29,30,31,32,33,34,35,36,37,38]. Alterations of anti-inflammatory properties and ability to promote cholesterol efflux have also been described in HDL treated in vitro with glucose [22]. Moreover, glycation triggered by glucose or MGO is associated with lipoprotein glyco-oxidation and impairment of HDL functions with a lower activity of the antioxidant enzyme PON1 [15,16]. Therefore, we thought of interest to investigate the effect of D-ribose on the activity of the enzyme HDL-paraoxonase 1 (HDL-PON 1), whose activity has been proposed as a useful laboratory test to measure HDL functions [48]. In fact, PON1 exerts a key role in HDL antioxidant and anti-inflammatory properties as shown in several studies [4,5,6]. A lower PON1 activity is widely described in dysfunctional HDL [4,7,8,9,10,11]. In the present study, the compositional changes in D-ribose-treated HDL are associated with a significant decrease in PON1 lactonase activity that is considered the PON1 native activity and contributes to the overall antioxidative function of HDL [49]. Alterations in functionality of D-ribose-treated HDL were confirmed by data from DHR-based cell-free assay which revealed a greater redox property of HDL treated with D-ribose. HDL redox capacity is a good marker of HDL functionality and is related to HDLs intrinsic ability to be oxidized as confirmed by the positive correlation established between DOR values and marker of lipid peroxidation evaluated in HDL treated in different experimental conditions. Functional HDL acts as a protective scavenger, and increased HDL redox activity indicates that HDL behaves as dysfunctional, pro-oxidant, and pro-inflammatory HDL. High HDL redox activity has been reported in pathological diseases such as coronary artery disease [50,51,52,53]. The significant negative correlation established between PON1 lactonase activity, HDL redox capacity and TBARS values observed in our experimental conditions confirms a role of lipid peroxidation on the enzyme activity and HDL functional properties [15,17,54,55].

We suggest that the loss of functional properties of D-ribose-glycated HDL may be likely related to the compositional changes in amino acid residues of Apo-AI and other proteins associated with HDL surface [56]. In fact, the increase in pentosidine and vesperlysine and total fluorescent AGEs demonstrate alterations of lysine and arginine residues of D-ribose-treated HDL. Other authors have shown that lysine glycation of Apo-AI impairs its anti-inflammatory function [57]. Lysine also exerts a crucial role for PON1 activity [58]. Modifications in residues of lysine and tryptophan appear also be involved in a reduced activity of HDL to remove cholesterol from the cells [59].

HDL antioxidant and anti-inflammatory properties have been widely described and recently reviewed [60]. It has been proposed that the anti-inflammatory role of HDL may be more significant than HDL cholesterol levels in the evaluation of risk prediction of cardiovascular disease [7]. Glycoxidation of HDLs triggered by glucose appears to modulate HDL anti-inflammatory functions. Even during chronic inflammation, reactive dicarbonyls like MGO can render HDL dysfunctional and pro-inflammatory.

The present study shows for the first time that even D-ribose contributes to compositional changes and alterations of HDL functions.

A limitation of this study is the use of relatively high D-ribose concentrations in the glycation experiments used in our experiments. In healthy individuals, D-ribose levels in blood and cerebrospinal fluid are typically around 0.01–0.1 mM [38], although higher levels have been reported in urine of diabetic patients [40,41,42]. Ribose exists both within and outside cells, and therefore this metabolite has the opportunity to glycate both intracellular and extracellular proteins. Protein modifications in vivo occur over extended periods via continual exposure to millimolar levels of D-ribose and the modifications induced by such exposure could likely to accumulate over time. In fact, the lifespan of HDL apolipoprotein, specifically Apo-AI, is about 2 to 4 days in circulation, during which HDL undergoes metabolic remodeling [61,62].

Despite this limitation, our findings could have physiological relevance and suggest that D-ribose-induced glycation of HDL may impair normal physiological functions and could play a role in the onset of a pro-inflammatory state and contribute to higher risk for cardiovascular disease in subjects with type 2 diabetes.

## 4. Materials and Methods

### 4.1. Separation and Glycation of HDL

Blood was sampled from 9 healthy subjects (5 females and 4 males, mean age of 42.5 ± 6.0 years) with normoglycaemic and normolipidaemic profiles, who had fasted overnight. Written informed consent was obtained from all participants before their enrollment in the study. The study was conducted in accordance with the principles outlined in the Declaration of Helsinki (1975, revised in 2013) and was approved by Ethical Committee of the “Azienda Ospedaliero-Universitaria Ospedali Riuniti” Ancona (Italy), Protocol number 211525, Approval Date: 21 December 2011 ensuring that the study adheres to both national and international guidelines. Serum was collected and stored at −80 °C until isolation of lipoproteins. HDL was isolated from a pool of serum samples by using precipitation of non-HDL particles with polyethylene glycol [63] and dialyzed at 4 °C for 24 h against 10 mM phosphate-buffered saline (PBS) solution (pH 7.4). Protein and cholesterol concentrations of HDL were determined by the method of Bradford [64] and by a colorimetric kit (Chema Diagnostica, Monsano, AN, Italy).

HDL (100 μg protein) was incubated in the absence or in the presence of different concentrations of D-ribose (20 mM, 40 mM and 80 mM) for different times (3 to 7 days) in a final volume of 1 mL of N_2_-saturated PBS containing 25 μM butylated hydroxytoluene (BHT), as previously described [25,29]. In some experiments HDL (100 μg protein) was incubated with methylglyoxal (MGO) (0.1 mM and 1.0 mM) in the same experimental conditions described above [17]. HDL incubated in the same experimental conditions without D-ribose or MGO was used as control (control HDL). At the end of the incubation, control HDL and HDL incubated with D-ribose or MGO were dialyzed against 0.9% NaCl solution at 4 °C for overnight.

### 4.2. Fluorescent Advanced Glycation End Products (AGEs)

The study of protein glycation triggered by different molecules is widely investigated using the evaluation of fluorescent advanced glycation end-products (AGEs). In fact, many AGEs such as pentosidine and vesperlysine exhibit fluorescent properties. Therefore, the presence of fluorogenic AGEs is commonly verified using fluorescence spectroscopy. To assess the formation of fluorescent AGEs, samples of control HDL and HDL treated with D-ribose or MGO, HDL samples were excited at different wavelengths. The increase in total AGE fluorescence intensity of treated samples was recorded using emission at (λexc 370 nm; λem = 450 nm) [17]. Specific AGEs were measured by fluorescence using characteristic excitation and emission wavelengths: vesperlysines-like (λexc 366 nm; λem 440 nm) and pentosidine-like (λexc 335 nm; λem 385 nm) [43,65]. Fluorescence emission spectra were recorded by a Perkin Elmer LS50B spectrofluorometer (Perkin Elmer, Rodgau, Germany).

### 4.3. Tryptophan Intrinsic Fluorescence

Intrinsic emission fluorescence spectra of tryptophan of control HDL and HDL treated with D-ribose or MGO were recorded using a Perkin Elmer LS50B spectrofluorometer using 295 nm as excitation wavelength [17].

### 4.4. Analysis of Hyperchromicity

The UV absorption measurements of control HDL and HDL incubated in the presence of D-ribose or MGO were obtained by measuring the ultraviolet absorption profile between the wavelength ranges of 200–800 nm using a Shimadzu spectrophotometer. (Shimadzu Corporation, Kyoto, Japan). The percentage increase in UV absorbance at 280 nm was evaluated.

### 4.5. Determination of Protein-Bound Carbonyl Groups

Evaluation of carbonyl groups in control HDL and in HDL treated with D-ribose for 7 days was estimated according to Levine et al. by using 2,4-dinitrophenylhydrazine (DNPH) [66]. The results were expressed as the number of nanomoles of carbonyl per mg of sample HDL using a ε379 nm = 22,000 M^−1^∙cm^−1^.

### 4.6. Thiobarbituric Acid-Reactive Substances (TBARS)

The extent of lipid peroxidation of control HDL and HDL treated with D-ribose for 7 days was evaluated by measuring the level of thiobarbituric acid-reactive substances (TBARS) as previously described [63].

### 4.7. HDL-PON Lactonase Activity

A volume (100 μg) of control HDL and of HDL treated with D-ribose for 7 days was resuspended in 50 mM Tris-HCl buffer (pH 7.5) with 1 mM CaCl_2_. After the addition of the substrate dihydrocoumarin (DHC) (1.0 mM), the hydrolysis was monitored at 270 nm for 5 min (every 15 s) using a Synergy HT microplate reader (BioTek, Winooski, VT, USA). One unit of lactonase activity was equivalent to one µmol of DHC hydrolyzed/min/mL [67].

### 4.8. Evaluation of Redox Capacity of HDL

A fluorometric biochemical cell-free assay was used to study the redox activity of untreated HDL and of D-ribose-treated HDL [50,68]. This protocol evaluates the effect of HDL on the oxidation rate of the substrate dihydrorhodamine 123 (DHR). A stock solution of DHR (50 mM) was diluted at 1:1000 in a hydroxyethylpiperazine-N-2-ethanesulfonic acid-buffered saline (HBS containing NaCl 150 mM, HEPES 20 mM, pH 7.4) as previously described. Samples of HDL (1.25 μg HDL-cholesterol) and DHR solution at a final concentration of 7 μM, were added in a 96-well plate. The volume was diluted to 200 μL with HBS buffer. A Synergy HT microplate reader (BioTek, Winooski, VT, USA) was used (485/538 nm excitation/emission filter). Fluorescence measurements were taken every 2 min over the course of 1 h. The DHR oxidation rate (DOR) was calculated for each well and was expressed as fluorescence units per minute (FU/min) as previously described [50,68].

### 4.9. Statistical Analysis

All data are expressed as mean ± standard deviation (SD). A GraphPad Prism 8.2 software (GraphPad, San Diego, CA, USA) was used to evaluate statistical analysis of data. Statistical significance of data was determined by Student’s *t* test, and a *p* value of <0.05 was considered as significant.

## Figures and Tables

**Table 1:** Total fluorescent AGEs, vesperlysine and pentosidine in HDL incubated in the absence (control HDL) or in the presence of increasing concentrations of D-ribose or methylglyoxal for 3 and 7 days. Data are reported as percentage increase in fluorescence emission intensity of treated HDL compared to control HDL. Data are mean ± standard deviation (SD) of three independent experiments. * *p* < 0.05, ** *p* < 0.01, *** *p* < 0.001, **** *p* < 0.0001 vs. control HDL.

	Total AGEs (%)		Vesperlysine (%)		Pentosidine (%)	
	3 Days	7 Days	3 Days	7 Days	3 Days	7 Days
**Control HDL**	100 ± 12	101 ± 14	100 ± 10	105 ± 16	100 ± 8	104 ± 13
**Ribose**						
20 mM	123 ± 22	153 ± 35 *	133 ± 18	185 ± 25 **	181 ± 26	250 ± 31 ***
40 mM	196 ± 18 ***	296 ± 18 ****	202 ± 25 ***	297 ± 21 ****	299 ± 29 ***	357 ± 32 ****
80 mM	253 ± 22 ***	383 ± 19 ****	258 ± 28 ***	340 ± 22 ****	330 ± 27 ****	414 ± 27 ****
**Methylglyoxal**						
0.1 mM	127 ± 20	195 ± 25 ***	-	-	-	-
1 mM	273 ± 14 ****	440 ± 10 ****	-	-	-	-

## Data Availability

Data will be made available on request.

## Abbreviations

The following abbreviations are used in this manuscript:

AGEs	Advanced glycation end products
Apo-AI	Apoprotein AI
CAD	Coronary artery disease
DHC	Dihydrocoumarin
DHR	123 dihydrorhodamine
DOR	DHR oxidation rate
HDL	High-density lipoprotein
LDL	Low-density lipoproteins
MGO	Methylglyoxal
PON1	Paraoxonase 1
TBARS	Thiobarbituric acid-reactive substances
TRP	Tryptophan
